# ULK4 in Neurodevelopmental and Neuropsychiatric Disorders

**DOI:** 10.3389/fcell.2022.873706

**Published:** 2022-04-12

**Authors:** Shilin Luo, Nanxi Zheng, Bing Lang

**Affiliations:** ^1^ Department of Pharmacy, The Second Xiangya Hospital, Central South University, Changsha, China; ^2^ Hunan Provincial Engineering Research Center of Translational Medicine and Innovative Drug, Changsha, China; ^3^ Department of Psychiatry, National Clinical Research Center for Mental Disorders, The Second Xiangya Hospital of Central South University, Changsha, China

**Keywords:** ULK4, neurodevelopmental disorder, neuropsychiatric disorder, pseudokinase, schizophrenia

## Abstract

The gene *Unc51-like kinase 4 (ULK4)* belongs to the *Unc-51-like* serine/threonine kinase family and is assumed to encode a pseudokinase with unclear function. Recently, emerging evidence has suggested that ULK4 may be etiologically involved in a spectrum of neuropsychiatric disorders including schizophrenia, but the underlying mechanism remains unaddressed. Here, we summarize the key findings of the structure and function of the ULK4 protein to provide comprehensive insights to better understand ULK4-related neurodevelopmental and neuropsychiatric disorders and to aid in the development of a ULK4-based therapeutic strategy.

## Introduction

Neuropsychiatric disorders are a wealth of debilitating brain diseases with overlapping etiologies, including genetic variants and environmental stress. The concordance rate is high and the heritability is substantial, although the influence of *de novo* mutations cannot be ignored especially in autism spectrum disorders (ASDs) (Alonso-Gonzalez et al., 2018). During the past decades, genome-wide association studies (GWASs) have reported numerous genetic alleles with single nucleotide polymorphisms (SNPs) (Uffelmann et al., 2021). In addition, recent progress in whole genome interrogation has also demonstrated massive genetic variants that are not covered by GWAS(Rao et al., 2021). The advances in research methodologies have expanded our understanding of the genetic architecture of psychiatric patients but also revealed further complexity. Hence, it is compelling to identify the predisposing risk alleles and to fully elucidate the associated mechanisms underpinning neuropsychiatric disorders. Unfortunately, thus far, only limited success has been achieved. Intriguingly, recent studies have revealed overwhelming evidence in neurodevelopmental elements in neuropsychiatric disorders (Cristino et al., 2014; Cardoso et al., 2019; Al-Naama et al., 2020). Various genetic alterations that occur during the embryonic stages can lead to pathological brain development and may precipitate the onset of psychosis in adolescence. These developmental insults are believed to disturb the neuronal connectivity and cellular architecture within the brain. The most common neurodevelopmental and neuropsychiatric disorders include depression, schizophrenia, autism spectrum disorders (ASD), bipolar disorder, attention deficit hyperactivity disorder, and X-linked intellectual disability, among others. The prevalence of these disorders is growing rapidly, which has caused a tremendous socioeconomic burden, primarily due to their high incidence in children and adolescents (Androutsos, 2012; Robertson et al., 2015; Hansen et al., 2018; Ghandour et al., 2019; Post and Grunze, 2021). During the past several decades, strenuous research has been performed in these fields. Unfortunately, the etiology and underlying mechanisms remain poorly understood.

In 2014, we first reported that *Unc-51-like kinase 4* (*ULK4*) is crucial for neuritogenesis and neuronal motility and, when defective, may predispose people to neuropsychiatric disorders including schizophrenia (Lang et al., 2014). Since then, accumulating evidence has strongly suggested that ULK4 participates in corticogenesis, cilia maintenance, myelination, and white matter integrity, although the precise downstream signaling pathways and interacting substrates remain elusive. Recently, we have provided evidence that ULK4 deletion can cause decreased intermediate neural progenitors and increased apoptosis, which strongly disrupt normal cortical development (Hu et al., 2021). In addition, ULK4 can form an interactome by physically binding with PP2A and PP1α, the two most abundant phosphatases, and is responsible for over 90% of total Ser/Thr dephosphorylation in eukaryotes. This interactome closely regulates the expression of p-Akt and p-GSK-3α/β, and mice with ULK4-targeted deletion in the excitatory neurons of the forebrain present a spectrum of core features of schizophrenia. These data collectively suggest that *ULK4* is a rare susceptibility gene for psychiatric disorders, especially schizophrenia. In this review, we will summarize the current knowledge of the roles of ULK4 in neurodevelopmental and neuropsychiatric disorders.

## Main Text

### Unc-51-like Serine/Threonine Kinase (ULK) Family

**FIGURE 1:**
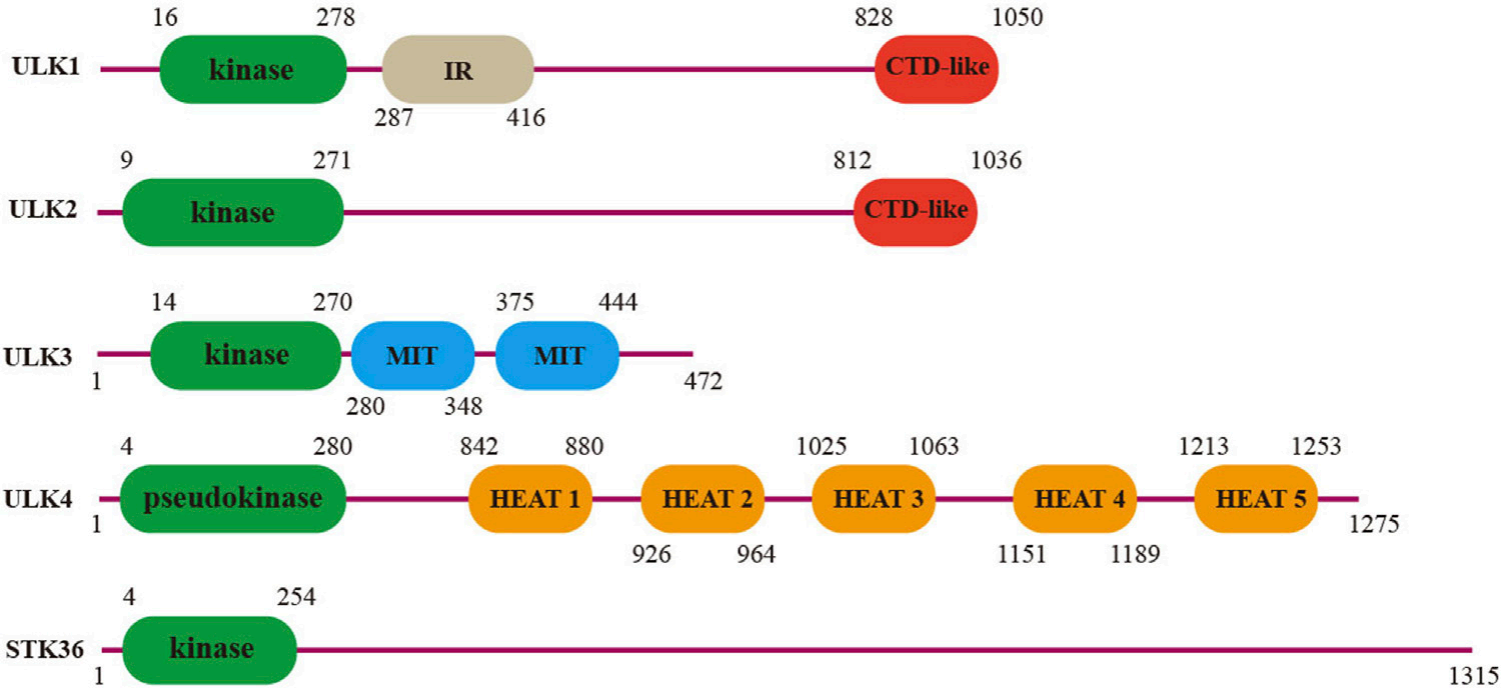
Domain architecture of the human ULK family. Protein interaction domains are annotated as interaction domain (IR), C-terminal domain (CTD) (ULK1 and ULK2), microtubule interacting, and trafficking molecule (MIT) (ULK3), and HEAT domains (ULK4).

In 1998, a novel mouse ortholog of the *Caenorhabditis elegans* serine/threonine kinase uncoordinated-51 (UNC-51) was first cloned (Yan et al., 1998), and thereafter, five related genes in total were found and grouped into the UNC-51-like serine/threonine kinase (ULK) family: ULK1, ULK2, ULK3, ULK4, and serine/threonine kinase 36 (STK36). The kinase domains of ULKs are conserved and located at the N-terminus, and the C-terminal region contains protein interaction motifs important for substrate recruitment (Figure 1). In mammals, ULK1 and ULK2 are evolutionarily conserved serine/threonine kinase orthologs of the yeast autophagy-related (ATG) family member ATG1, and play a necessary but somewhat redundant function in proper autophagy initiation (Wang et al., 2018). The high-resolution structure analysis shows that ULK1 and ULK2 share a high degree of conservative domain architecture, including an N-terminal catalytic kinase, extensive middle linker, and C-terminal domain essential for interaction with their binding partners (Lazarus et al., 2015; Chaikuad et al., 2019). During autophagy, the canonical early regulatory complex consists of ULK1/ULK2, ATG13, RB1-inducible coiled-coil protein 1 (RB1CC1, also known as FIP200), and ATG101, which translate upstream nutrient and energy signals (e.g., mTOR and AMPK) into the downstream autophagy pathway (Ganley et al., 2009; Jung et al., 2009; Wong et al., 2013; Lin and Hurley, 2016). Disrupting ULK1 expression in mice leads to defective autophagy-mediated clearance of mitochondria, and mice lacking both ULK1 and ULK2 die shortly after birth due to a defect in glycogen metabolism, which is similar to what occurs with other autophagy-defective mice (Kundu et al., 2008; Cheong et al., 2014). Apart from these processes, ULK1/ULK2 also regulates TrkA receptor trafficking and signaling, which instructs filopodia extension and neurite branching during sensory axon outgrowth (Zhou et al., 2007). Knockdown of ULK2 reduced asymmetric neuropil elaboration and affected habenular development in the brain (Taylor et al., 2011). Recently, *Kang et al.* revealed an association between ULK2 polymorphisms and schizophrenia in the Korean population (Kang et al., 2022).

The other three homologs, ULK3, ULK4, and STK36, contain kinase domains homologous to ULK1/2 but do not have a conserved C-terminal sequence, and they participate in many physiological processes to maintain tissue homeostasis. ULK3 has been reported to be involved in the autophagy induction during senescence (Young et al., 2009). It also has a dual function in the Sonic hedgehog signal transduction pathway, which controls a variety of developmental processes and is implicated in tissue homeostasis and neurogenesis in adults (Fuccillo et al., 2006; Maloverjan et al., 2010). STK36 is essential for the central pair apparatus and cilia orientation of motile cilia in mice. The cilia of STK36^−/−^ mice are stiff and exhibit significantly reduced stroke amplitude or even immotile movement, which eventually promotes the occurrence of hydrocephalus (Merchant et al., 2005; Nozawa et al., 2013). According to the database in the Swiss Institute of Bioinformatics (SIB), ULK4 is widely expressed in different systems, especially in the secretion system, immune system, and nervous system, but its precise function remains largely unclear. Since we first reported that ULK4 may be a rare susceptibility gene for schizophrenia in 2014, research on this gene has been springing up in the neuropsychiatric field.

### ULK4 Protein Structure

ULK4 is a large protein (142 kDa) encoded by the gene *Unc51-Like Kinase 4*, which is located on human chromosome 3p22.1 (Went et al., 2019). Unlike the homolog family member ULK1-3, the ULK4 protein contains a pseudokinase domain at the N-terminus and is thus predicted to be catalytically inactive. There are five HEAT repeats at the C-terminus of ULK4 (842–880, 926–964, 1,025–1,063, 1,151–1,189, and 1,213–1,253) (Figure 1), which are commonly found in large proteins, such as mTOR, and are presumably involved in protein scaffolding or interaction (Andrade et al., 2001; Perry and Kleckner, 2003). The crystallized high-resolution structure of ULK4, including its small-molecule inhibitor and ULK4-ATP-rS, has been recently interpreted by two independent research groups (Khamrui et al., 2020; Preuss et al., 2020). Notably, ULK4 can bind to ATP in an unusual Mg^2+^-independent manner, and the affinity is higher than that of any known pseudokinase (Khamrui et al., 2020). Because some pseudokinases are capable of binding to ATP and allosterically regulating the catalytic functions of kinases using compensatory motifs, even though ULK4 has no apparent phosphotransferase activity (Zeqiraj and van Aalten, 2010), it is assumed that like many others, ULK4 may work as the sensor of ATP and undergo conformational changes upon the binding which subsequently promotes its roles as a scaffold for substrate recruitment. Indeed, *Preuss et al.* predicted many ULK4 interacting partners including active kinases and phosphatases, which require further functional validation (Preuss et al., 2020).

Similar to the working mechanism of STRAD/LKB1, the pseudokinase domain of ULK4 specifically interacts with STK36. This strongly indicates that ULK4 can regulate active kinases directly, despite it being deemed catalytically inactive (Zeqiraj et al., 2009). The unique C-terminal HEAT repeats may enable ULK4 to bind to proper substrates or interacting proteins using a similar recruitment mechanism as ULK1/2. This hypothesis was further substantiated by Preuss and his colleagues, who have revealed that these repeated regions interacted uniquely with calmodulin-regulated spectrin-associated protein 1 (CAMSAP1), oral-facial-digital syndrome 1 (OFD1), and poly(A)-specific ribonuclease subunit 2 (PAN2) (Preuss et al., 2020). However, thus far, there has not been any report that there is an interaction partner of the ULK4 HEAT repeats at the C-terminal of STK36. Domain mapping of ULK4 provides a structural framework for its roles in diseases.

### ULK4 and *Unc-51*

The *unc-51* gene was initially described in the nematode *C. elegans* by Brenner in 1974 and showed extensive expression during embryonic brain development when neurons were actively extending their axons, particularly in the head region of late embryos (Brenner, 1974). Surprisingly, worms with the *unc-51* mutation were mostly paralyzed, egg-laying defective, and dumpy (McIntire et al., 1992; Ogura et al., 1994). These data strongly suggested that the unc-51 protein is essential for axon maintenance and elongation. In the brains of *Drosophila* individuals, unc-51-mediated membrane vesicle transport is pivotal in the targeted localization of guidance molecules and organelles that regulate the elongation and compartmentalization of developing neurons as well as motor-cargo assembly (Mochizuki et al., 2011). Similarly, the unc-51 protein was reported to localize in the vesicular structures of growth cones of cerebellar granule cells and spinal sensory neurons in mice, which controls axon formation in granule cells through the endocytic membrane trafficking pathway (Tomoda et al., 1999; Tomoda et al., 2004). As a homologous serine/threonine kinase of unc-51 in humans, ULK4 was initially reported to be associated with blood pressure and hypertension (Levy et al., 2009; Ehret and Caulfield, 2013; Konigorski et al., 2014). Meanwhile, it may be involved in cell cycle control, as its polymorphisms (rs1052501 and rs2272007) were associated with multiple myelomas (Broderick et al., 2011; Greenberg et al., 2013). Inspired by the physiological functions of unc-51, we reanalyzed the common and rare variants of ULK4 in the databases of the International Schizophrenia Consortium (ISC) and among the bipolar Icelandic cases genotyped by deCODE Genetics, and we discovered that it may serve as a rare susceptibility gene for human mental disorders, especially schizophrenia (Lang et al., 2014). Our subsequent functional study further revealed that ULK4 is involved in the remodeling of cytoskeletal components, such as acetylation of *α*-tubulin, and in this way regulates neurite branching and elongation as well as cell motility.

### ULK4 and Neurogenesis

Both *in vivo* and *in vitro* studies have suggested that ULK4 may play a key role in neurogenesis and corticogenesis during developmental stages. In *Xenopus* embryos, ULK4 mRNA is mostly expressed in the ventricular (VZ) and subventricular zones (SVZ) zones and distributed throughout the brain after the closure of the neural tube. Constant expression of ULK4 has also been found in neural stem cells in adult *Xenopus* (Domínguez et al., 2015). Similarly, Ulk4 transcripts are widely found in the VZ, SVZ, and cortical plate in the E15.5 cortex in mice, and ULK4 protein is widely expressed in all cortical layers after postnatal Day 7. Knockdown of ULK4 at E15.5 significantly inhibited cell proliferation and corticogenesis in mice (Lang et al., 2016). Meanwhile, the size of the neural stem cell pool in the forebrain that is important for adult neurogenesis was remarkably reduced in ULK4 null knockout mice at birth (Liu et al., 2016a). Although normal cortical lamination was preserved, the knockout mice showed a thinner cortex due to defective cell proliferation. As abnormal neurogenesis is often associated with neurodevelopmental or neuropsychiatric diseases (Kang et al., 2016; Guarnieri et al., 2018), it is therefore believed that ULK4 may contribute to the development of these diseases. *Liu et al.* further identified that ULK4 expression was dependent on the cell cycle, with a peak expression in the G2/M phases, and it decreased during both embryonic and adult neurogenesis in ULK4 mutant mice, probably because of a dysregulated Wnt signaling pathway (Liu et al., 2017).

### ULK4 and Neurite Arborization

It has been well documented that Unc-51 regulates the dendritic development in the brains of individuals of the genus *Drosophila* through kinesin-mediated membrane transport (Mochizuki et al., 2011). In *C. elegans*, Unc-51 mutation often leads to abnormal axonal elongation and structures (Ogura et al., 1994). Consistently, appropriate neurite arborization is important in establishing synaptic connectivity and neuronal plasticity, which is critical for preventing the onset of schizophrenia (Mochizuki et al., 2011; Mizutani et al., 2019). Therefore, it is assumed that the ULK family plays an important role in the establishment of the appropriate neural network and, when defective, may promote the development of neurological diseases. In line with this hypothesis, our data suggest that the proper expression of ULK4 is critical for neurite branching and brain development. Knockdown of ULK4 in SH-SY5Y cells led to less expression of acetylated *α*-tubulin, which may underlie the reduced dendrite length and/or branching and compromised neuronal migration (Lang et al., 2014). Defective neuritogenesis may involve multiple signaling pathways including protein kinase C (PKC), mitogen-activated protein kinase (MAPK), extracellular signal-regulated kinase (ERK), and c-Jun N-terminal kinases (JNK) (Lang et al., 2014). Similarly, our *in utero* electroporation study *in utero* also demonstrated that knockdown of ULK4 caused perturbed neurite arborization in the pyramidal neurons of the cortex (Lang et al., 2016).

### ULK4 and the Integrity of White Matter

Children’s performance in cognition, intelligence, processing speed, and problem solving is closely associated with the thickness of the white matter, such as the corpus callosum and defective myelination is a hallmark related to neurodevelopmental and neuropsychiatric disorders (Liu et al., 2018b). We previously showed that ULK4 null knockout mice displayed impaired genesis of the corpus callosum (Lang et al., 2014). *Liu et al.* further reported a 50% decrease in myelination in ULK4^−/−^ mice together with a general reduction in myelin components (Liu et al., 2018b). Myelin is produced by oligodendrocytes and controls impulse conduction speed along the axon, which is important to cognitive performance. Children with a less myelinated white matter in their brains often display developmental delay problems. Meanwhile, ULK4 mutant mice also present thin axons and extensive neuroinflammation, which also promote the occurrence of hypomyelination. In addition, ULK4 deficiency significantly attenuated the enrichment of oligodendrocyte transcription factors, the newly formed oligodendrocytes, and myelinating oligodendrocytes (Liu et al., 2018b). These data collectively indicate that ULK4 may be a crucial factor for the integrity of white matter and myelin.

### ULK4 and Ciliopathy

The cilium is an antenna-like structure that protrudes from the surface of almost all mammalian cells. It participates in multiple signaling transduction pathways and when defective, can result in a series of inherited disorders called “ciliopathies”. The most common features of ciliopathy include cystic liver and/or kidney, blindness, neural tube defects, brain anomalies, mental disability, skeletal abnormalities, obesity, and infertility, among others (Oud et al., 2017). Genomic and bioinformatics research has revealed that some primary cilia genes are linked to psychiatric disorders, such as the genes *CC2D2A* and *Disc1*, which are involved in ciliogenesis (Shen et al., 2008; Marley and von Zastrow, 2010; Veleri et al., 2014), and their defects can lead to psychiatric disorders, including Joubert syndrome (Bachmann-Gagescu et al., 2012), mental retardation (Noor et al., 2008; Shi et al., 2012), Meckel syndrome (Tallila et al., 2008), and Bardet Biedl syndrome (BBS) (Haq et al., 2019). In addition, several signaling pathways and crucial factors highly associated with schizophrenia, such as Wnt signaling, the fibroblast growth factor signaling system, neuronal migration, and the dopamine hypothesis, are dependent on the complete functionality of the cilium, although the specific mechanism is not yet well understood (Marley and von Zastrow, 2010; Muraki and Tanigaki, 2015; Narla et al., 2017; Hoseth et al., 2018). In the mouse brain, ULK4 is strongly expressed in the choroid plexus and ependymal cells lining the ventricles (Lang et al., 2014). Both ULK4 null knockout and hypomorphic mice present disturbed motile cilia development and disorganized ciliary beating which impair CSF flow and eventually lead to congenital hydrocephalus (Vogel et al., 2012; Liu et al., 2016b). These data strongly indicate the potential connection between ULK4 haploinsufficiency and ciliopathy. Acetylated *α*-tubulin is an important cytoskeletal component of cilia that is instrumental for cilium assembly. Our study, however, revealed that knockdown of ULK4 in human neuroblastoma cells (SH-SY5Y) and the mouse brain led to reduced expression of acetylated *α*-tubulin (Lang et al., 2014; Lang et al., 2016). In addition, whole-genome RNA sequencing also revealed massive disruption of genes closely related to ciliogenesis including Foxj1, Pcm1, Tubb4a, Dnah9, Rsph4a, Gsn, Kif5a, Lgals3, Lgals3bp, and Dnal1 in ULK4 mice carrying hypomorphic alleles. Interestingly, it has been reported that Foxj1 may target downstream substrates including Spag6, Rsph9, Rsph4a, Dnah9, Dnal1, Ttll6, and Tekt2 which consequently impairs ciliary development and results in hydrocephalus (Liu et al., 2016b). A recent study also reported that patients with a microdeletion of the *ULK4* gene and a microduplication of the *BRWD3* gene manifested core features of ciliopathy such as psychomotor delay, epilepsy, autistic features, hearing loss, obesity, minor facial dysmorphisms, peculiar ear malformations, and skeletal abnormalities (such as dorsal kyphosis and/or valgus knees and flat feet) (Tassano et al., 2018). Thus, it is highly likely that ULK4 contributes to ciliopathies. The results demonstrate that ULK4 is crucial for ciliogenesis and ciliopathies.

### The Progress of Current Research on ULK4 in Mental Disorders

**FIGURE 2:**
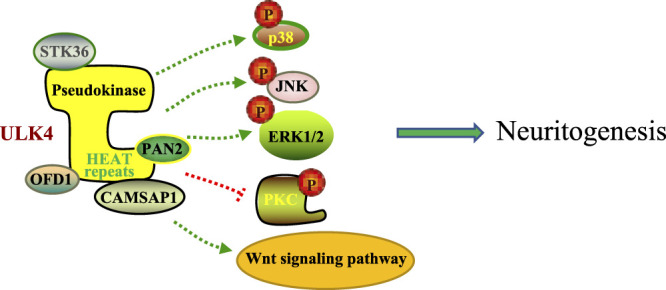
A schematic representation of altered activities of multiple signaling pathways including p38 MAPK, JNK, ERK1/2, PKC, and Wnt signaling pathways by ULK4. These alterations contribute to deficient neuritogenesis, a common feature frequently represented by human mental disorders.

Although previous GWAS studies have suggested that ULK4 is a risk locus for multiple myeloma and interindividual diastolic blood pressure variation, emerging evidence also supports the idea that *ULK4* genetic variants may cosegregate people with multiple neuropsychiatric disorders (Levy et al., 2009; Broderick et al., 2011). In our previous research using the cohort data from the International Schizophrenia Consortium, we identified four schizophrenia patients with *ULK4* intragenic fragment deletions spanning from exon 21 to exon 34 among 3,391 schizophrenia patients (Lang et al., 2014). Another study implicated that SNPs rs7651623 and rs2030431 of *ULK4* are associated with the risk of discontinuing the use of antipsychotics in patients with schizophrenia (Ou et al., 2019). In the Decode database, ULK4 deletion was also enriched in patients with schizophrenia (2/708), bipolar disorder (2/1,136), and autism (1/507) (Lang et al., 2014). In addition, association signals were observed at SNPs rs1052501, rs1716975, and rs2272007, which are located in exons 2, 7, and 17 of *ULK4*, respectively, for allelic transmission disequilibrium from parents to their children with ASD (Ou et al., 2019). Similarly, SNP rs17210774 of *ULK4* is significantly associated with bipolar disorder in Caucasians and another SNP rs1722850, which is close to but downstream of *ULK4,* is related to major depressive disorders (Lang et al., 2014) (Table 1). A recent study of the brain-body genetic resource exchange (BBGRE) cohort also reported an incidence in a population of 1.2‰, showing ULK4 copy number variation and exhibiting pleiotropic neurodevelopmental problems including learning difficulties and language delay (Liu et al., 2016a). In addition, a recent clinical study revealed 2 cases with ULK4 intragenic microdeletion (together with partial microduplication of BRWD3) that showed autistic features (Tassano et al., 2018).Consistently, in the follow-up functional analysis, we have revealed that knockdown of *ULK4* altered the activity of Wnt, PKC, MAPK, ERK1/2, and JNK signaling pathways commonly found in human mental disorders, especially schizophrenia (Figure 2). In addition, both *ULK4* knockout and hypomorphic mice presented congenital hydrocephalus featuring dilated ventricles and CSF accumulation. Interestingly, a proportion of schizophrenia patients also display increased global or regional CSF(Vogel et al., 2012; Lang et al., 2014). Moreover, *Liu et al.* revealed that ULK4 heterozygous mice displayed anxiety-like behavior with reduced GABAergic neurons in the basolateral amygdala and hippocampus (Liu et al., 2018a), and ULK4^−/−^ mice showed a significant hypomyelination phenotype (Liu et al., 2018b). All these studies strongly suggest that ULK4 may be a rare risk factor for neuropsychiatric disorders including schizophrenia but more evidence is warranted in the future.

**TABLE 1:** Summary of ULK4 variants and relevant manifestation in human patients.

SO Term	Ref Allele	Alt Allele	SNP Number	Related Disease	Ref
intron	C	T	rs17210774	bipolar disorder	Lang et al. (2014)
intron	T	C	rs1722850	depressive disorder	Lang et al. (2014)
5 UTR	A	G	rs7651623	risk of discontinuing use of antipsychotic medications in the patients with schizophrenia	Ou et al. (2019)
intron	C	T	rs2030431	risk of discontinuing use of antipsychotic medications in the patients with schizophrenia	Ou et al. (2019)
missense (A542P/A542T)	C	G/T	rs1052501	ASD/multiple myeloma	(Broderick et al., 2011; Greenberg et al., 2013)
missense (K39R/K39T)	T	G/C	rs2272007	ASD/multiple myeloma	Ou et al. (2019)
intron	T	A/C	rs1717027	diastolic blood pressure	Franceschini et al. (2013)
missense (I224F/I224V)	T	A/C	rs1716975	ASD	Ou et al. (2019)
intron	T	G	rs4973978	ASD	
intron	T	C	rs9824775	ASD	
intron	T	C	rs6599175	ASD	
intron	G	A	rs6783612	ASD	
intron	C	T	rs9852303	ASD	
intron	A	G	rs4973893	ASD	
intron	T	C	rs1716670	ASD	

## Conclusion and Perspectives

Although ULK4 is a member of the Unc-51-like kinase family, unlike its ortholog members ULK1-3 and STK36, it is predicted to be catalytically inactive and to function as a pseudokinase. Initially, ULK4 was found to be associated with blood pressure and hypertension but further research has indicated its important functions during neurodevelopment. Knockdown of ULK4 *in vitro* also altered the activities of multiple signaling pathways, including Wnt, PKC, p38 MAPK, ERK1/2, and JNK, and mice with ULK4 deletion showed anxiety-like behaviors, perturbed neurogenesis, and decreased myelination. As mentioned above, ULK4 may be a rare risk factor for a range of psychiatric disorders, including schizophrenia, ASD, bipolar disorder, and depression, whose genetic variants were found in relevant patients and are crucial for ciliogenesis and ciliopathies. Further studies are warranted to fully understand the important function of ULK4, especially in neurodevelopment, and the specific underlying mechanisms for psychiatric disorders. With the successful resolution of the protein structure of ULK4 and further elucidation of its function, a series of small molecules targeting ULK4 may be developed to alleviate relevant neurodevelopmental and neuropsychiatric disorders in the future.
